# Undergraduate scientific education and the decline of postgraduate medical researchers in Germany? A perspective discussion and review of the literature

**DOI:** 10.3389/fmed.2025.1639839

**Published:** 2025-08-11

**Authors:** Philipp Stieger, Alexander Peter Schwoerer, Holger Buggenhagen, Rüdiger C. Braun-Dullaeus, Christian Albert

**Affiliations:** ^1^Otto-von-Guericke University Magdeburg, University Clinic for Cardiology and Angiology, Magdeburg, Germany; ^2^Department of Cellular and Integrative Physiology, University Medical Centre Hamburg-Eppendorf, Hamburg, Germany; ^3^Department of Anesthesiology, Rudolf Frey Teaching Clinic, University Medical Center Mainz, Johannes Gutenberg University Mainz, Mainz, Germany; ^4^Department of Nephrology, Central Clinic Bad Berka, Bad Berka, Germany

**Keywords:** scientific competence, scientific thinking, scientific working, scientific acting, medical education, postgraduate education, doctorate, thesis

## Abstract

The decline of postgraduate medical researchers in Germany reflects fundamental gaps in undergraduate scientific education. Scientific competence (SC)—the integrated ability to think, act, and work scientifically—underpins the academic pipeline, yet its curricular implementation in frameworks such as NKLM 2.0 (National Competency-Based Learning Objectives Catalogue Medicine) and the revised ÄApprO (Approbationsordnung für Ärzte, German Licensing regulations for doctors), remains vague. German curricula do not distinguish Stokes’s “knowledge” vs. “utility” dimensions of research, nor Kölbl’s pedagogical refinements, reducing SC to a technical adjunct rather than an inquiry-driven competence vital for clinical decision-making. Student surveys reveal broad appreciation for scientific thinking but report scant structural support for independent research. Without clear, multidimensional learning objectives and longitudinal embedding—via modular research projects, protected research time, and continuous mentorship—interest in research-oriented careers will continue to wane. We call for SC to be redefined as a core, practice-integrated pillar of medical training, transcending its current role as a curricular checkbox and securing the future of academic medicine.

## Introduction

### Physician shortage and their associated consequences

Germany’s healthcare sector has experienced a growing shortage of board-certified physicians in recent years, a trend mirrored internationally (1). This shortage stems from demographic trends and workforce attrition, but also reflects systemic weaknesses in medical education—particularly in how future physicians are trained in both clinical practice and scientific inquiry.

Based on a nationwide survey of 30 German medical schools covering 33 degree programs (response rate 83%), in which only 21% had a dedicated evaluation system and just 46% conducted in-course assessments, nearly half of final-year students still report feeling unprepared for independent practice—exposing critical gaps not only in clinical training but also in the structured development and quality assurance of physician-scientist competences (2, 3). At the root of this issue lies a crucial but underdeveloped element of medical education: the integration of “scientific competence” (SC) into the curriculum. These gaps in both clinical training and research preparation stem from curricula that lack explicit SC targets.

The consequence is clear: fewer young professionals pursue careers in medical research, shrinking the pool of postgraduate investigators and threatening both patient care and the future of clinical science—a trend documented not only in Germany but also in international contexts (4–7).

To reverse these trends, German medical curricula must more rigorously define and embed SC learning objectives—articulating clear, measurable outcomes that span the critical appraisal of evidence, research design, and translational application—so that undergraduate training can strengthen clinical capabilities while cultivating the next generation of medical researchers.

**Figure 1:**
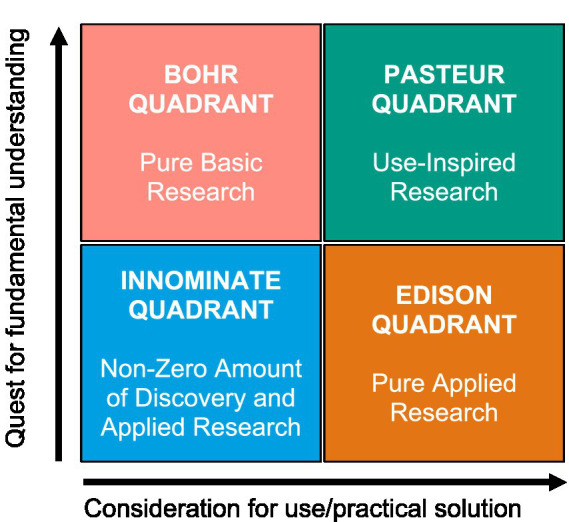
Model maps research by its pursuit of understanding and practical use, distinguishing pure basic (Bohr), applied (Edison), and use-inspired basic research (Pasteur)—highlighting the dual aim essential for scientific competence (SC) (9).

Before evaluating curricular challenges and reform needs, we introduce the theoretical foundation of SC. Following the conceptual model by Stokes (8), scientific activity can be mapped alongside two axes: a quest for understanding (knowledge dimension) and a consideration for use (utility dimension) (8). While basic research typically aims at theoretical insight [e.g., Niels Bohr (1885–1962)—fundamental, with his study on atomic theory and nuclear physics], applied research is primarily oriented toward practical solutions [e.g., Thomas Edison (1847–1931)—practical, with his invention of the light bulb] (Figure 1). Crucially, use-inspired basic research—as exemplified by Louis Pasteur—demonstrates how scientific inquiry can simultaneously advance fundamental knowledge and address real-world problems.

Ideally, scientific competence in medical education should reflect both dimensions—the ability to understand scientific principles and to apply them in clinical and translational settings. Despite this solid framework, these dual dimensions are only partially integrated into German curricula, limiting practical application.

Building on this framework, Kölbl (10) transferred these dimensions into higher education pedagogy, describing scientific competence as a multidimensional construct consisting of knowledge-based, action-oriented, and design-related sub-competencies (10). In the medical context, these include:

- “Scientific thinking” (methodological understanding, critical reasoning; knowledge-oriented),
- “Scientific working” (executing research projects, applying methods; utility-oriented),
- “Scientific acting” (designing research, generating own questions; knowledge-driven with creative intent).

### Formal recognition of scientific competence and its “double omission”

Germany’s National Competency-Based Learning Objectives Catalogue for Medicine (NKLM 2.0) and the forthcoming revision of the medical licensure regulation (Approbationsordnung für Ärzte, ÄApprO) formally include SC as a core training outcome. While these developments mark critical policy-level recognition of SC, their impact remains limited by a lack of operational detail.

Yet these frameworks stop short of operationalizing SC’s two indispensable dimensions: a rigorous pursuit of fundamental understanding (“knowledge dimension”) and a purposeful translation into clinical practice (“utility dimension”). This dual shortcoming—the absence of both theoretical depth and practical application—creates a disconnect between policy intention and curricular implementation. This double omission of both theory and application stands in striking contrast to leading North American Competency-Based Medical Education (CBME) initiatives—such as Canada’s Competence by Design (CBD) and the U. S. LCME’s Entrustable Professional Activities (EPAs)—which explicitly define, map, and assess both dimensions, yielding measurable gains in trainee research engagement and clinical confidence (11). Recognizing this gap, it becomes imperative to examine how the lack of defined SC dimensions affects learners and the broader academic pipeline.

### Curricular ambiguity and its impact on the academic pipeline

Having established the theoretical framework and policy context, we go on to examine how curricular ambiguity affects learner trajectories and the academic pipeline. In Germany, curricular ambiguity around SC contributes to wide inter-institutional variability in graduate skill sets (12), undermines targeted instruction, and erodes the pipeline of future physician-scientists. Medical studies in Germany typically span 6 years and 3 months. They are divided into three main phases: a pre-clinical phase (“Vorklinik,” 2 years), a clinical phase (“Klinik,” 3 years), and a final practical year (“Praktisches Jahr” or PJ, 1 year). There are exactly three state examinations (“Staatsexamina”)—the First Section (M1 or “Physikum”) after the pre-clinical phase, the Second Section (M2) after the clinical phase, and the Third Section (M3) after PJ. Upon passing all three exams, the graduates can apply for the license to practice medicine (“Approbation”). An academic doctoral degree (“Dr. med.”) is optional and usually earned through an independent research project and dissertation, typically conducted during or after medical school. Postgraduate specialty training (e.g., in internal medicine, surgery, or general practice) is regulated by the regional medical chambers and takes an average of 5–8 years, depending on the discipline and specific training regulations for specializations (13). Early-career clinicians in university centers often describe the “triple burden” of patient care, research, and teaching—exacerbated by a curriculum that fails to foster SC as both inquiry and application—as a deterrent to academic careers (14–16). A recent mixed-methods study of Germany’s clinical clerkship (Famulatur) found that, without explicit learning objectives, students feel insecure and overly dependent on the clinical team—an uncertainty that, when carried through subsequent training phases, further impedes progression into research-oriented specialist careers (17).

## Scope of this perspective and a vision for reform

To address the intertwined challenges of physician shortage, inconsistent research preparedness, and curricular ambiguity, this Perspective critically examines the current status of SC in German medical education. Thus, in this Perspective, we first dissect the curricular gap in the definition and assessment of scientific competence within German medical education, highlighting how the absence of clearly differentiated knowledge and utility dimensions undermines both teaching and evaluation. We then draw actionable lessons from established international CBME exemplars—such as Canada’s Competence by Design and the U. S. LCME’s EPAs—to illustrate how explicit mapping of competences to milestones and assessments can drive measurable improvements in trainee research engagement and clinical confidence. Finally, we propose concrete, scalable reforms—including the introduction of EPA-based milestones at each training phase, the development of shared digital CBME platforms for workplace-based assessments and e-portfolios, and robust faculty development programs—to reinvigorate scientific training across all stages of German medical education. Through this integrated approach, our goal is to move beyond problem description toward a cohesive framework for reform. By unifying both the knowledge and utility dimensions of scientific competence within a competency-based framework, Germany can standardize graduate outcomes, strengthen its pipeline of physician-scientists, and ultimately enhance the quality and safety of patient care.

However, despite this well-established theoretical foundation, current educational frameworks in German medical education rarely distinguish or systematically incorporate these dimensions. Neither the NKLM 2.0 nor current drafts of the new ÄApprO provide separate learning objectives for knowledge-oriented vs. utility-oriented scientific competencies. As a result, students often perceive scientific competence merely as a technical skill for clinical practice, not as a pathway to academic inquiry or autonomous research. This lack of conceptual clarity in the curriculum contributes to an underdeveloped perception of SC, reducing it to procedural knowledge rather than fostering it as a longitudinal academic trajectory. While the conceptual differentiation between knowledge- and utility-oriented scientific competence draws on established theoretical frameworks by Stokes (8) and Kölbl (10), one might question whether this model resonates beyond academic discourse and is accepted within the broader German medical education community. In fact, recent national initiatives suggest that these ideas are gaining institutional traction: the updated NKLM 2.0 explicitly defines “scientific competence” as a key graduate attribute, and the forthcoming revision of the ÄApprO echoes this multidimensional approach (3, 18). Moreover, position papers by the German Council of Science and Humanities (Wissenschaftsrat, WR) and policy frameworks such as the “Masterplan Medizinstudium 2020” increasingly reflect a shared understanding that scientific competence must encompass both the capacity for critical inquiry and its practical application in care delivery (19). These developments indicate growing alignment between educational policy and the multidimensional concept of SC, even if full curricular operationalization remains a work in progress.

Thus, while not yet universally operationalized, the dual conception of scientific competence articulated here is aligned with national reform goals and is increasingly accepted among medical faculties and policymakers. Building on this policy alignment, it remains critical to explore how these curricular frameworks translate into actual student experiences and perceptions. To this end, we conducted empirical investigations to assess medical students’ understanding of scientific competence and its role in their education and career aspirations.

## Students’ perceptions and experience: empirical insights

In this regard, based on competency-based education, the requirements of the curriculum and the expectations of those involved in the learning process are of relevance (20, 21).

Accordingly, in an online survey of 339 medical students recruited via internal mailing lists and voluntary lecture announcements, our group recently interviewed students at three university locations (University of Hamburg): *n* = 162, Otto-von-Guericke University, Magdeburg: *n* = 97, Johannes Gutenberg University, Mainz: (*n* = 80). Participants were recruited via internal mailing lists and voluntary lecture announcements. The inclusion of students from three different medical faculties allowed for a broad representation across both preclinical and clinical phases of medical education. However, the generalizability of the findings to all German medical students may be limited due to potential institutional differences in curriculum design and research culture.

**Figure 2:**
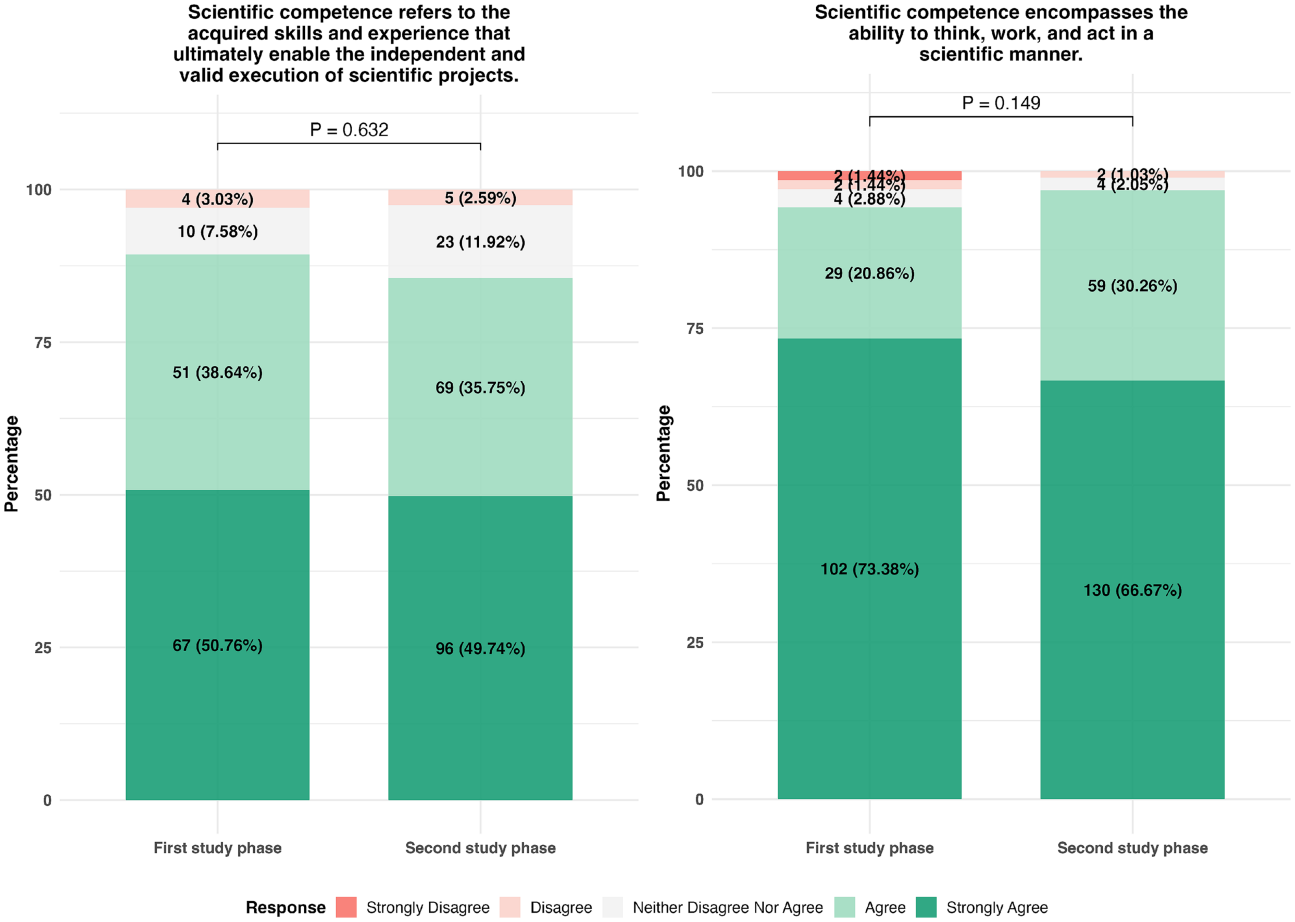
Assessment of students’ perception regarding “scientific competence” based on a 5-point Likert scale. Participants responded on a scale from “strongly agree” to “strongly disagree” (1 = strongly agree, 2 = agree, 3 = neither agree nor disagree, 4 = disagree, and 5 = strongly disagree). Response rates were 95.87% (*N* = 325) and 98.52% (*N* = 334), respectively. Group comparisons were conducted using the chi-squared test with the R Environment for Statistical Computing (The R Foundation, Vienna, Austria). A *p* < 0.05 was considered significant.

We found that, in both the early and later phases of their medical studies, the students, despite the likely presence of self-selection bias typical for convenience sampling, had very clear and, above all, uniform ideas about the definition of SC. For the vast majority of respondents, and with increasing agreement as students gained more experience, SC was described as the “acquisition of skills and experiences that ultimately enabled them to pursue and successfully carry out independent scientific projects” and that for doing so SC as an adjunct to the “ability to think, work and act in a scientific manner” was needed (Figure 2). This definition, rooted in both knowledge and application, aligns with the dual-dimensional model of scientific competence and highlights students’ intuitive grasp of its multifaceted nature. From the students’ perspective, medical doctors require SC regardless of whether they would perform in research or clinical practice.

These findings highlight a key disconnect within German medical education: while the majority of medical students—even in the early study phases—report a surprisingly coherent and experience-dependent understanding of SC and explicitly consider it relevant for both clinical and research roles, only a small fraction actually continues into structured research careers. This gap between perceived importance and later engagement highlights the need for curricula that not only define SC within the abovementioned framework but also enable its longitudinal development and application.

**Figure 3:**
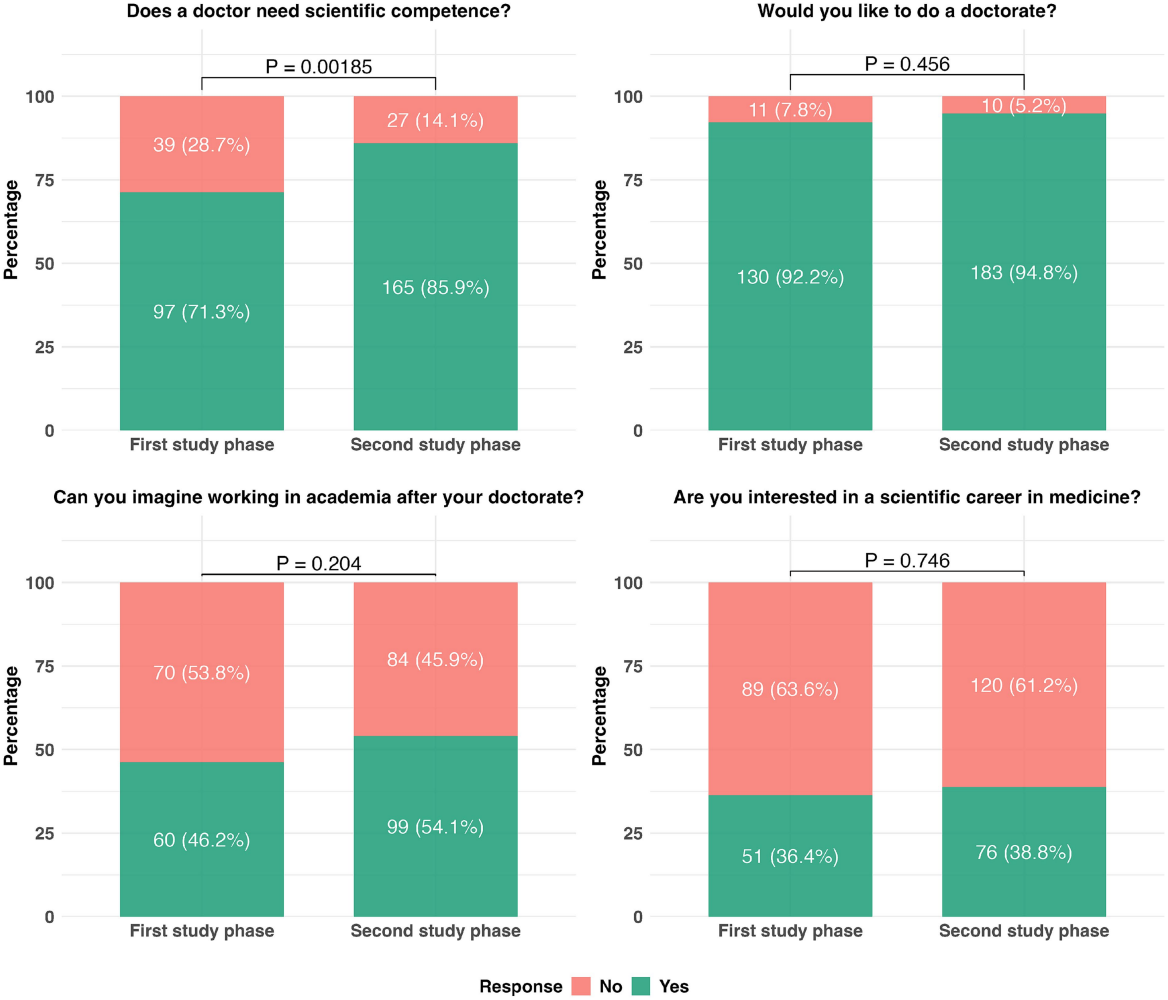
Assessment of students’ motivation to pursue a doctorate or a scientific career. Response rates were 96.76 (*N* = 328), 98.53% (*N* = 334), 92.33% (*N* = 313), and 99.12% (*N* = 336), respectively. Group comparisons were conducted using the chi-square test with the R Environment for Statistical Computing (The R Foundation, Vienna, Austria). A *p* < 0.05 was considered significant. The questionnaire was developed de novo and piloted with a small group of students to ensure clarity and relevance, but no formal validation procedures were performed, given the exploratory purpose of this illustrative data and the convenience of sample size determination.

While this Perspective focuses on educational levers to support academic career interest, it is important to acknowledge that broader systemic factors—such as the structural challenges of residency training, including service load, lack of protected research time, and the “triple burden”—may also significantly limit the translation of student interest into long-term academic engagement. In this context, we found that at the beginning of medical students’ studies, approximately 70% of respondents were interested in pursuing a doctoral project and thesis, while at the end of their training, even more students obtained this interest (Figure 3). This is complemented by further data: regardless of study phase, over 90% of respondents indicated a general intention to pursue a doctorate (Figure 3), and at the time of the survey, 70% were already planning a specific scientific project (data not shown). As expected, this proportion was higher among students in the latter half of their studies (81 vs. 54%, *p* < 0.0001). These high levels of interest suggest that motivation alone is not the limiting factor—rather, the lack of structured, supportive training environments may be responsible for the attrition of future physician-scientists.

Independent of study progress, about half of the surveyed students could envision engaging in academia after the doctorate, while 38% expressed interest in a general scientific career in medicine (Figure 3)—a motivation further reinforced by targeted, high-engagement experiences such as international electives (22). Despite this, students repeatedly expressed a lack of adequate guidance and support, citing insufficient preparation for research-related tasks and a lack of continuity in developing SC.

However, students expressed a lack of adequate support for these goals, reflected in free-text responses such as: *“I am at the beginning of my doctoral research and have to laboriously figure everything out by myself,”* and *“Never having written a term paper or engaged independently and scientifically with a topic, the skills that would enable students to work scientifically—perhaps once acquired at school—are becoming somewhat buried.”*

In contrast, a 2011 graduate survey showed that while students described their medical training as fundamentally scientific and research-oriented, only a very small proportion were interested in the methodology behind research outcomes. These findings reinforce the theoretical distinction made by Stokes (8) and Kölbl (10): although students may conceptually differentiate between the application-oriented and knowledge-driven dimensions of science, this distinction is often blurred in practical training contexts. Students reported receiving sufficient preparation for SC through projects lasting only 2–6 weeks or, at most, 10 weeks—despite most having completed the majority of their degree programs. This reveals a persistent mismatch between student expectations, the design of scientific training, and the longitudinal integration demanded by frameworks such as NKLM (2.0) and the German Council of Science and Humanities (Wissenschaftsrat, WR) recommendations.

Even though guidelines from the WR define SC as a necessary skill to apply scientific evidence in complex care situations, students did not perceive a need for longitudinal structures or translational concepts (such as the physician as a “lifelong learner”) at any point in their studies (24).

Thus, from a curricular perspective, this empirical insight highlights a crucial gap: while scientific thinking and working are valued, the structural conditions supporting independent research capacity are insufficiently developed for cultivating students’ independent research capacity and academic identity. This aligns with the previously stated concerns that SC is often reduced to technical competence for clinical practice, rather than cultivated as an academic or research trajectory (25).

## Discussion

The ongoing discussion about SC in medical education in Germany has gained increased relevance due to the structural shortage of specialist doctors and the declining number of research-oriented physicians. While political and curricular reforms (e.g., NKLM 2.0, the long-anticipated but still pending revised ÄApprO) explicitly aim to promote scientific skills, and initiatives such as DFG (Deutsche Forschungsgesellschaft, German Research Society) research grants for students and the establishment of Excellence Clusters have begun to incentivize early engagement in research, current empirical studies and student reports reveal a profound mismatch between aspirational goals and educational reality (26). The persistent increase in workload caused by clinical responsibilities and the lack of structured postgraduate training paths significantly undermines the scientific motivation of young doctors. The “triple burden” of clinical work, teaching, and research leads to research being perceived more as an additional stressor rather than as an integral part of the professional identity at university centers. This perception causes many young doctors to pursue more accessible, practice-oriented career paths, which in turn contributes to the erosion of interest in academic medicine (12).

Despite normative progress, the curricular integration of SC remains conceptually and didactically confusing. The NKLM 2.0 and current drafts of the new ÄApprO fail to systematically distinguish between knowledge-based and application-oriented competencies, as theorized by Kölbl (10) and Stokes (8). This ambiguity often leads to SC being understood narrowly as a technical skill for clinical problem-solving rather than as the foundation for critical reflection or independent scientific development. Empirical findings show that students recognize the general importance of SC but lack lasting opportunities and structural support for engaging in research beyond short-term project work (27). A more detailed discussion of the distinction between knowledge-based and application-oriented competencies would clarify how these concepts could strengthen SC in practice. For instance, Kölbl emphasizes the importance of not only acquiring scientific facts but fostering scientific reasoning, while Stokes’ framework differentiates between pure and use-inspired research, both relevant to the design of medical curricula.

Another key issue is the lack of curricular visibility and accessible role models for scientific inquiry. The observed decline in students’ interest in pursuing a doctoral thesis reflects a broader disconnect between the ideal of research-oriented medicine and the actual educational experience. Although many students begin their studies with a willingness to engage in research, this enthusiasm diminishes over time due to insufficient institutional support and the absence of meaningful research pathways embedded within the curriculum (20, 28). In addition, although medical students increasingly value scientific competence, only a minority go on to pursue research careers. This gap stems from the perceived challenges of academia—such as job insecurity, excessive bureaucracy, and limited mentorship—when contrasted with more stable and appealing alternatives in clinical practice. In this context, valuing scientific skills does not necessarily translate into a desire to follow a scientific or academic career path.

### The practical barriers to reform implementation

While these challenges underscore the urgency for reform, practical obstacles to implementation deserve attention. For example, integrating mandatory research pathways within an already demanding and overloaded medical curriculum poses significant organizational and financial challenges. It remains unclear how medical faculties can balance clinical training, teaching obligations, and research activities without exacerbating student and faculty workload. Addressing these barriers is crucial to the feasibility and sustainability of the proposed reforms.

### International CBME models as conceptual frameworks

To further support our conceptual distinction, we point to international developments in CBME that have gained increasing relevance in German reform debates. For instance, Canada’s Competence by Design framework (29) and the U. S. LCME’s Entrustable Professional Activities (EPAs) (11), both of which explicitly integrate and assess knowledge-related and application-related components of scientific competence, serve as models available for operationalizing such multidimensional constructs. These frameworks not only validate the distinction that we propose but also offer implementation pathways that could inform the adaptation of CBME in the German context. We therefore argue that articulating scientific competence along these two dimensions may contribute to conceptual clarity, and in the process, support the ongoing curricular reforms with both international best practices and the practical realities of undergraduate medical training in Germany.

### Scholarly concentration tracks and longitudinal research programs

Our findings resonate with international data showing that mandatory, longitudinal Scholarly Concentration programs substantially increase medical students’ research productivity. A systematic review of U. S. Scholarly Concentration tracks revealed that participants produced significantly more peer-reviewed articles and conference abstracts compared with students in traditional curricula, with strong mentorship and administrative infrastructure driving these gains (23). Similarly, interviewees in our study advocate for modular, multi-semester research frameworks that allow SC to develop continuously rather than in isolated projects. However, a critical reflection on the transferability of such evidence to the German context is still warranted, considering differences in educational systems, cultural expectations, and funding structures.

### The case for structured research pathways in Germany

In the United States, medical schools have increasingly implemented “Scholarly Concentrations”—structured research pathways often beginning in the third year of medical school—in response to broader educational trends and evolving accreditation expectations. While the Liaison Committee on Medical Education (LCME) does not explicitly mandate such programs, its accreditation standards, particularly since their 2015 revision, emphasize the importance of fostering an environment that supports student research and scholarly inquiry [e.g., Standard 3.2 (30)]. As a result, many institutions have adopted longitudinal research tracks to demonstrate compliance and enhance students’ scientific engagement. In contrast, Germany currently lacks mandatory, structured research programs within its medical curricula, despite a stronger emphasis on the “Scholar” role in the NKLM 2.0 and the revised ÄApprO. As part of the federal initiative “Masterplan Medizinstudium 2020” (31), expert commissions have recommended a comprehensive restructuring of medical education and an amendment to the ÄApprO, explicitly emphasizing the need to strengthen scientific competencies. Encouragingly, some German medical faculties have begun piloting structured research tracks or integrating mandatory scientific projects, signaling a shift toward more longitudinal and competency-based scientific training. Building on these emerging models may offer a feasible path for broader implementation.

Introducing a requirement similar to the LCME standards could help ensure that research training becomes a compulsory and integral part of medical education in Germany, rather than remaining optional.

### Combined degree programs as career pathways

Combined MD–PhD programs in countries such as the USA and the UK (e.g., Johns Hopkins University and Imperial College London) integrate research training with clinical education, creating clear career trajectories for physician-scientists (32, 33). Implementing similar pathways in Germany could reform our doctoral culture and foster research interest during undergraduate studies by providing formalized research time and mentorship (34).

### Global health curricula as a broader model of SC

The Global Health curricula also offer a model for integrating SC with broader societal issues. At the University of Oxford, a Global Health module combines evidence-based medicine with health equity research, involving students in international study projects (35). Introducing an equivalent program in German medical schools would broaden SC to include intercultural competencies and health service research, preparing graduates for global medical challenges.

### Longitudinal EBM training to promote critical thinking

Finally, longitudinal evidence-based medicine (EBM) seminars have been shown in Canada and the US to enhance critical appraisal skills and promote an academic mindset rather than purely clinical application (36, 37). Our participants viewed EBM training as the key to embedding SC as an enduring scholarly attitude. Integrating continuous EBM modules into the curriculum would reinforce both critical thinking and lifelong learning, aligning German medical education with international best practices.

### From interest to implementation: addressing the structural gap

These findings highlight a persistent gap: although many students—already in early study phases—express a coherent understanding of SC and recognize its relevance for both clinical and academic roles, only a small proportion pursue structured research careers (38). National statistics reflect that while over 60% (39) of medical students complete a doctoral degree (34, 40), fewer than 5% engage in long-term clinical research or enter clinician–scientist programs (38, 40). Our results point toward one-third of students being highly interested in science, yet only approximately 10% envision conducting research projects themselves.

This discrepancy suggests that early motivation is not matched by structural opportunities. In response, the DFG and the German Council of Science and Humanities have recommended training 5–8% of residents in German university hospitals as clinician-scientists (approximately 110–180 annually) (40). This provides a realistic benchmark for curricular and policy initiatives. Future reforms should thus focus not only on defining SC but on enabling its longitudinal development through structured support and integrated academic training pathways.

## Conclusion

Conceptualizing SC as a multidimensional educational objective—embedded throughout the entire curriculum and supported by clearly defined, operationalized learning objectives—would represent a decisive step toward revitalizing interest in academic medical careers. Integrating knowledge generation and practical application across all phases of training, along with the establishment of protected research time, mentoring, and translational learning environments, could foster a new generation of physician-scientists. International comparisons emphasize that while Germany has set normative frameworks such as the NKLM and the awaited revised ÄApprO, its practical curricular development currently lags behind countries with established “Scholarly Concentrations” or MD–PhD programs. A stronger adaptation of best-practice models—such as modular research project units, mandatory peer-review workshops, and interdisciplinary Global Health initiatives—could help close the gap between curricular objectives and student experience. Ultimately, scientific competence should be more than an abstract curricular goal–it should be practiced, visible, and actively cultivated within the realities of medical education.

## Data Availability

The original contributions presented in the study are included in the article/supplementary material, further inquiries can be directed to the corresponding author.
